# Quantitative insights into televised birth: a content analysis of *One Born Every Minute*

**DOI:** 10.1080/15295036.2018.1516046

**Published:** 2018-10-04

**Authors:** Sara De Benedictis, Catherine Johnson, Julie Roberts, Helen Spiby

**Affiliations:** a Department of Social and Political Science, Brunel University London, London, UK; b Department of Culture, Film and Media, University of Nottingham, Nottingham, UK; c Division of Midwifery, University of Nottingham, Nottingham, UK

**Keywords:** Birth, midwifery, content analysis, *One Born Every Minute*, reality television, medicalization

## Abstract

This article explores birth representations through a content analysis of two seasons of the U.K. program, *One Born Every Minute* (*OBEM*) (Channel 4, 2010–). Reality television (RTV) has been a fertile ground for the mediation of birth, but has also stoked controversy among feminist critics and the birth community about how birth is represented and the impacts this might have for women and society. International research has explored problematic over-representation of white, heterosexual couples, as well as noting a predominance of medicalized birth experiences. However, this research is formed largely of qualitative studies that are necessarily based on small samples of episodes. To contribute to this literature, we apply a quantitative and interdisciplinary lens through a content analysis of two seasons of the U.K. version of *OBEM*. Paying attention to the geographical and temporal context of *OBEM*, this article confirms over-representation of white, heterosexual couples and medicalized birth on RTV birth shows while also providing novel insights into the ambiguous representation of birthplace and lead caregivers, the medicalization of birth through the routinization of supposedly minor birth interventions, and the absence of the representation of women’s choice over such interventions.

Over the past two decades, western culture has seen a rise in birth representations, often mediated through reality television (RTV) (Tyler & Baraitser, 2013). Such representations, especially in British and American contexts, have attracted scholarly attention because they raise important issues around the politics of representation and of maternity care. However, this emerging body of literature mainly features close qualitative readings of RTV that make compelling claims about the politics of televised birth but necessarily focus on a small number of scenes or episodes, therefore making it difficult to fully support such claims or to generalize. This article aims to address the relative absence of quantitative research into televised birth with a content analysis of two seasons of the British series, *One Born Every Minute* (*OBEM*) (Channel 4, 2010–). We interject into the growing corpus of qualitative scholarship concerning how birth is televised by supporting, extending and challenging existing analyses as well as providing additional insights.

Because different temporal and national contexts actively shape normative representations of televised birth (Bull, 2016; Horeck, 2016), we limit our analysis to the U.K., examining birth representations within this specific maternity care context. The U.K. has a mixed-model of maternity care from midwives and obstetricians. Women can give birth at home; in an obstetric unit; an alongside midwifery unit or freestanding midwifery unit. The latter two sites are referred to as midwifery units (MUs). Clinical guidelines published by the National Institute for Health and Care Excellence (NICE) provide evidence-based information to guide National Health Service (NHS) maternity care for childbearing women with various medical, maternity or social needs. Guidance covers topics like birthplace, involving women in care decisions, assessing the well-being of women and their babies, labor progress and pain relief methods.

We begin by examining qualitative studies that have explored televised birth, with a particular focus on those relevant to the U.K. context, reflecting on and justifying the case study chosen. We detail the methodology used before presenting the data from a content analysis of two seasons of *OBEM*. Our focus is the representation of women who give birth, the birthing practices and procedures depicted, and how choice and information over such practices and procedures are portrayed. Through this analysis, we ask questions about how birth is represented on television in dialogue with existing international literature on RTV and contemporary debates around U.K. maternity care.

## Exploring birth representations

Tyler and Baraitser (2013) argue that while birth has become visible across a range of media, birth representations proliferate on television. Birth is the focus of U.K. RTV docusoaps (e.g., *Five Star Babies*, B.B.C. Two, 2016), dramas (e.g. *In the Club*, B.B.C. One 2014–) and soap operas (e.g., *EastEnders*, B.B.C. One, 1985–). RTV’s claim to represent reality and ability to make visible important (yet often hidden) social experiences makes birth an ideal topic for RTV (De Benedictis, 2017). There have been some U.K. studies that consider scripted television shows (Clement, 1997; Kitzinger & Kitzinger, 2001), with more recent studies comparing *Call the Midwife* (B.B.C. One, 2012–) alongside RTV (Hamad, 2016) or documentary (Takeshita, 2017). Yet, RTV is a distinct genre and has received the majority of scholarly attention (e.g., Bull, 2016; Horeck, 2016; Jackson, Land, & Holmes, 2017; Siebert, 2012; Tyler & Baraitser, 2013) as televised birth has flourished.

Existing research on birth and U.K. RTV highlights that even though “the taboo of childbirth is being broken as birth is becoming routinely witnessed and represented in more graphic and public ways” (Tyler & Baraitser, 2013, p. 1), this is often in troubling manners. Feminists investigating U.K. RTV have raised concerns about the dramatized, medicalized nature of televised birth and the representations of women’s birthing bodies as lacking, with technology and pain relief positioned as able to resolve this inferiority (Tyler & Baraitser, 2013). Birth is understood in this literature as placed in increasingly medicalized and masculinized frameworks, echoing broader social ideologies of birth (Feasey, 2012). Tyler and Baraitser (2013, p. 9) argue “childbirth TV” shows like *OBEM* represent women as “passive subjects” in accordance with localized medical practices in the Global North that reify “control” and “surveillance” of women’s bodies. Feminists have argued that in U.K. RTV (and scripted television; Clement, 1997; Kitzinger & Kitzinger, 2001), birth is presented as a dangerous emergency that medical professionals bring under control (Tyler & Baraitser, 2013). Scholars have investigated and questioned the extent to which *OBEM* in particular depicts women as having choice and control over birth (Jackson et al., 2017; O’Brien Hill, 2014). However, some studies also note positive potential for RTV birth shows. Feasey argues *OBEM* “can be seen to offer more realistic and thus potentially informative image of contemporary hospitalised childbirth” (Feasey, 2012, pp. 174–175).

Local and national specificity brings the politics of time and place to considerations of birth on RTV. Horeck (2016) compares the U.K. and U.S. versions of *OBEM.* She argues that the healthcare systems represented in *OBEM* U.K. (midwifery-led state healthcare) and *OBEM* U.S. (private obstetric healthcare) shape how birth is represented. *OBEM* U.K. represents relatively diverse births, e.g., water births and assisted birth, whereas *OBEM* U.S. largely depicts medicalized birth through induction and epidurals. Horeck asserts that these differing representations circulate birth norms according to localized contexts and they position the viewer to react to birth opposingly, the former in more empathetic ways and the latter in more distanced ways. By contrast, O’Brien Hill (2014), in exploring the representation of the “older” birthing mother in *OBEM* U.K., argues that the representation of “natural” birth in *OBEM* is framed by broader discourses of control in birth that (re)create the “good” mother myth through an investment in “natural” birth. She questions how much choice women really have during birth if one choice is seen as superior and if the midwife is represented as the authority (see also Siebert, 2012). This argument is partly supported by Jackson et al.’s (2017) conversation analysis of *OBEM* U.K. which found that midwives and obstetricians are represented as asserting decisions in risky and routine birth activities, rather than partaking in shared decision-making practices with women. However, the division between the “natural” and “medical” model of birth, and women’s choices within these frameworks, are not always clear-cut. Through comparing U.K., U.S. and Scandinavian shows, Bull (2016) argues that some U.K. and Scandinavian birthing shows blur these models (see also Siebert, 2012). Bull (2016, p. 187) asserts that *OBEM* U.K. depicts various birth behaviors (e.g., vocalizations) associated with the “natural” model of birth, alongside representation of “the midwife as both a skilled medical professional and a distinctly female figure of care” (opposing negative midwifery representation in U.S. shows). Thus, she complicates the notion of the “natural” and “medical” models of birth being mutually exclusive to argue that the value of “natural” birth is nonetheless upheld in such shows.

Explorations of birth in U.S. RTV programs have focused more explicitly than the U.K. literature on questions of diversity. For example, studies highlight that RTV birth representations in *A Baby Story* (Discovery Channel/TLC, 1998–2011) offer normative depictions, whereby the nuclear family is upheld as the bedrock of American society by depicting largely heterosexual, familial narratives (Morris & McInerney, 2010; Sears & Godderis, 2011; Stephens, 2004). However, there has been work in the U.K. context that has addressed diversity largely through examination of the classed dimensions of the birthing subject. This scholarship has explored the ridiculing of working-class mothers as “bad” birthing subjects (De Benedictis, 2017; Siebert, 2012) and the differing classed depictions of how working and middle-class women achieve control during birth (O’Brien Hill, 2014). In various ways, in both the U.K. and the U.S., then, diversity has emerged as a core issue in the representations of birthing subjects in RTV.

The literature explored above offers strong qualitative insights into birth on U.K. RTV. However, to date, there is a paucity of quantitative evidence. The only U.K. quantitative study is Clement’s (1997) content analysis of 154 British factual and fictional programs that featured birth. This is the closest to our study but was published over 20 years ago. As such, its findings relate to a very different historical context. Clement (1997, p. 40) argues that birth is depicted as “fast, dramatic and unpredictable,” in hospitals, surrounded by doctors, with little pain relief or intervention in the first stage of labor and as a subsequent risky crisis for mother or baby in the second stage of labor before a safe birth. She notes that these representations neglect slow, quotidian births. However, whilst valuable, this content analysis was performed in 1993 and predates RTV’s rapid explosion. More recently, Morris and McInerney (2010) have undertaken content analysis of 123 representations of birth on 85 RTV shows in the U.S. context. They highlight that birth was not depicted without medical intervention and that there was an over-representation of heterosexual and married women. These two studies suggest the value of content analysis. They quantify the types of births and women that are represented, and they underscore the repetition and volume of limited imaginings of televised birth. For example, Morris and McInerney (2010) found that breech birth was over-represented compared with actual U.S. rates in their data set, and those breech births that were delivered vaginally were depicted as dramatic, medical emergencies, findings not apparent in qualitative studies of RTV. This is why multiple methods are necessary to explore the mediation of birth; while qualitative research can identify types of representation, quantitative methods can index their prevalence.

Finally, we note that the scholarship detailed above mainly coalesces in media studies or sociology. Despite RTV birth representations being of concern elsewhere, such as midwifery (see Roberts, De Benedictis, & Spiby, 2017), little research speaks across disciplines, with one recent exception (Luce et al., 2016). Thus, the exploration of birth on U.K. RTV would benefit from an interdisciplinary approach to offer quantitative insights that consider the localized, temporal contexts within which these representations operate.

## Methodology

Content analysis aims to offer a systematic analysis of a cultural phenomenon (Messenger Davies, & Mosdell, 2006, p. 98). Content analysis can provide statistical evidence of which births, procedures and women are represented, and any correlations between these. Content analysis can be contextualized against existing qualitative studies, providing statistical data to corroborate (or challenge) concerns about what kinds of experiences of birth are (or are not) depicted, which is central to the analysis of the broader sociocultural understandings of the nature of childbirth.^1^

The content analysis presented here explores seasons eight and nine of the primetime, British Academy of Film and Television Arts (BAFTA) Award-winning television show, *OBEM*. The show places over 40 cameras in U.K. maternity wards. *OBEM* positions itself as a documentary, although it is heavily edited and often sensationalized, following RTV’s generic conventions (De Benedictis, 2017). *OBEM* is the longest running and most popular U.K. birth show. At the time of writing in May 2018, the eleventh season is airing and the series has consistently retained strong audience numbers; Hamad (2016) notes that until 2014 the series regularly commanded approximately three million viewers. Although season ten premiered with a dip in viewership at just over two million viewers (BARB, 2017), *OBEM* still regularly featured in the ten most watched programs in the weeks it aired on Channel 4. Furthermore, *OBEM* has garnered considerable attention from the birth community because of its claim to truthfully represent birth and concerns about its potential impact on women (J. Roberts et al., 2017). Because of the show’s popularity and the surrounding debates, it is a productive example to consider televised birth. Additionally, recent qualitative studies of televised birth in the U.K. and beyond (e.g. Horeck, 2016; O’Brien Hill, 2014; Siebert, 2012) have focused on *OBEM* and offer useful context against which the findings of this study can be situated and interrogated.

Seasons eight and nine of *OBEM* were picked as they were both filmed in Liverpool Women’s Hospital, airing over a 1-year period.^2^ This offered a reasonable sample size to explore, located in a similar geographical and policy context. In these two seasons, three births were shown per hour-long episode and there were 16 episodes in total, therefore producing a sample size of 48 births (*n* = 48), enabling us to undertake statistical analysis within the available timeframe.

The co-authors collaboratively devised the coding categories. The team represents disciplinary perspectives from cultural and media studies, sociology of health and midwifery. The aim to bring an interdisciplinary focus was derived from the wider project in which this analysis is located. *Televising Childbirth*, funded by the Wellcome Trust, combines theoretical insights from these disciplines to better understand the significance of televised birth and its impact on women’s health. The content analysis codes were directed by the politics of representation, birth and maternity care in the U.K., and we addressed a series of interrelated questions about: how birth was represented on television, which social groups were given visibility through such representations, how such representations relate to the medicalization of birth (Oakley, 1984), women’s autonomy and labor choices (Malacrida & Boulton, 2014), as well as midwifery’s struggles for professional status within the NHS (Mander & Murphy-Lawless, 2013).

To speak to our concerns about which women were depicted giving birth on *OBEM*, we created codes around class, race and ethnicity, sexuality, disability and relationship status. Drawing on Orgad and De Benedictis (2015) and Skeggs, Thumim, and Wood (2008), class was subcategorized into “upper class,” “middle-class,” “lower middle-class/working-class” and “unclear/unknown.” With Van Sterkenburg, Knoppers, and De Leeuw’s (2010) critique of the propensity for content analyses to delimit subcategorizations of race and ethnicity to “black–white,” we informed subcategories by the social context that media discourses are embedded. We used subcategories of “white,” “black,” “Asian,” “minority ethnic” and “unclear/unknown” reflecting contemporary U.K. diversity discourses, although we acknowledge that the “black, Asian and minority ethnic” (BAME) categorization is problematic as it still works to define whiteness (Saha, 2017). Considering Barker and Langdridge’s (2008) critique of the tendency to silence some sexualities in research, we subcategorized sexuality into “heterosexual,” “homosexual,” “bisexual,” “other” and “unclear/unknown”. Drawing on Briant, Watson, and Philo’s (2013) classifications, disability was subcategorized into “physical/sensory,” “mental health,” “learning disability,” and “no disability.” Lastly, relationship was subcategorized into “couple,” “couple (partner absent),” “single,” “single (partner present),”^3^ “other” and “unclear/unknown”. To analyze the representation of the medicalization of birth, subcategories were created drawing on midwifery expertise in the team to explore the depiction of birth method, birth location, procedures during birth, pain relief during birth, birth position (first stage of labor), birth position (second stage of labor) and lead caregiver. For emergency cesareans, position of the second stage of labor was coded as “not applicable.” For scheduled cesareans, overall the position at the first and second stages of labor were coded as “not applicable,” but there were occasions when a woman entered the first stage of labor and then had a scheduled caesarean, and in these cases the birth position of the first stage of labor was coded. To analyze how choice and information over these procedures were depicted, subcategories were created to explore the representation of information, choice and outcome over procedures and information, choice and outcome over pain relief. We coded what appeared on *OBEM* following these categories, but not the airtime each category received.

De Benedictis and Roberts performed a pilot analysis of nine episodes in total. After consultation, the research team amended categories to produce a final coding framework. De Benedictis coded all items, and Roberts and Spiby double coded 15% and 5% respectively. The team discussed and resolved any discrepancies that arose from double coding prior to inputting the data into SPSS. Frequencies and cross-tabulations were run on the data and points of interest were discussed by the co-authors to inform the analysis. The following sections explore the results.

## Representing contemporary mothers: whose births are televised?

As we have seen above, questions of diversity have emerged as a central concern in the U.S. and U.K. literature about representations of birth in RTV. Scholars have argued that the soothing tales of the human drama of a newborn arriving in the world are usually only available to protagonists in heterosexual, two-parent families (De Benedictis, 2017; Morris & McInerney, 2010; Stephens, 2004) that are often white (Sears & Godderis, 2011) and sometimes middle-class (Siebert, 2012). In general, our research confirmed concerns about the lack of diversity in representations of birthing women in RTV. Women represented in the two seasons were primarily white (96%), heterosexual (98%), in a relationship (83%) and able-bodied (92%). This largely accords with Morris and McInerney’s (2010) content analysis of American birthing shows, although they found more racial diversity.

Ahmed (2010, p. 45) argues that the family is a “happy object;” it “is both a myth of happiness, of where and how happiness takes place, and a powerful legislative device.” The representations of predominately heterosexual, white, able-bodied couples depicted as the subjects of birth stories are problematic and limiting, and come to (re)create normative conceptions of birth and family life in these two seasons of *OBEM*.

**Figure 1.**
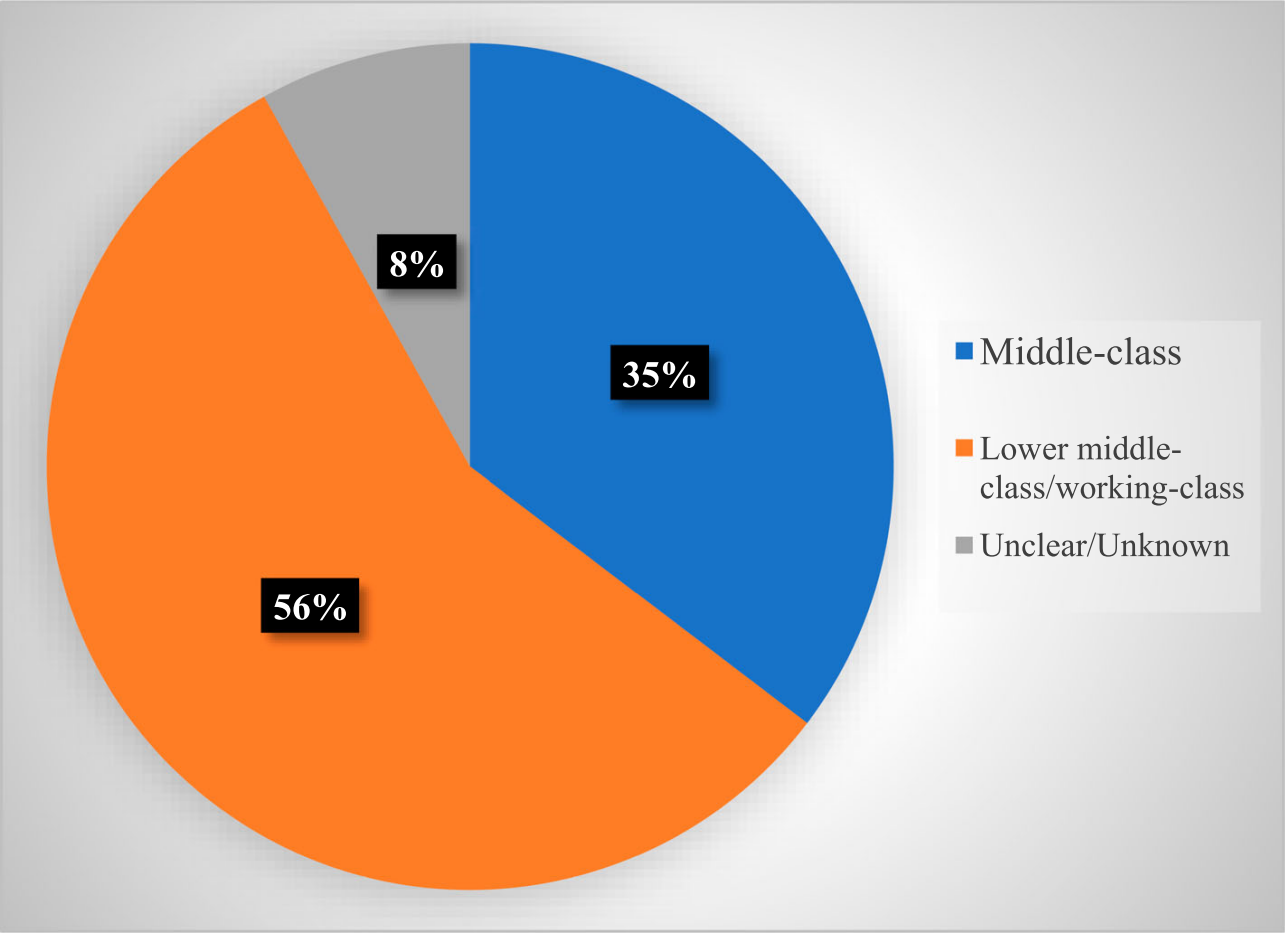
Class. Note: percentages may not add up to 100% on figures due to rounding up or down.

Considering the wider literature on gender, class and reality television, we might expect to see a vast over-representation of lower middle/working-class women, however, in accordance with some qualitative studies of *OBEM* (Feasey, 2012; O’Brien Hill, 2014)*,* we found the class composition of women in *OBEM* to be relatively mixed. Some 56% of birthing women were depicted as lower middle/working-class, 35% as middle-class and 8% had “unclear/unknown” class backgrounds (Figure 1). This supports existing scholarship about class and RTV. A number of scholars note a propensity for the genre to parade the unpaid labor of lower middle/working-class women (Skeggs & Wood, 2012; Tyler, 2011). Around a third of women in *OBEM*, however, were depicted as middle-class, highlighting women of different class backgrounds are represented giving birth, albeit the majority of women were still depicted as lower middle/working-class women.

**Figure 2.**
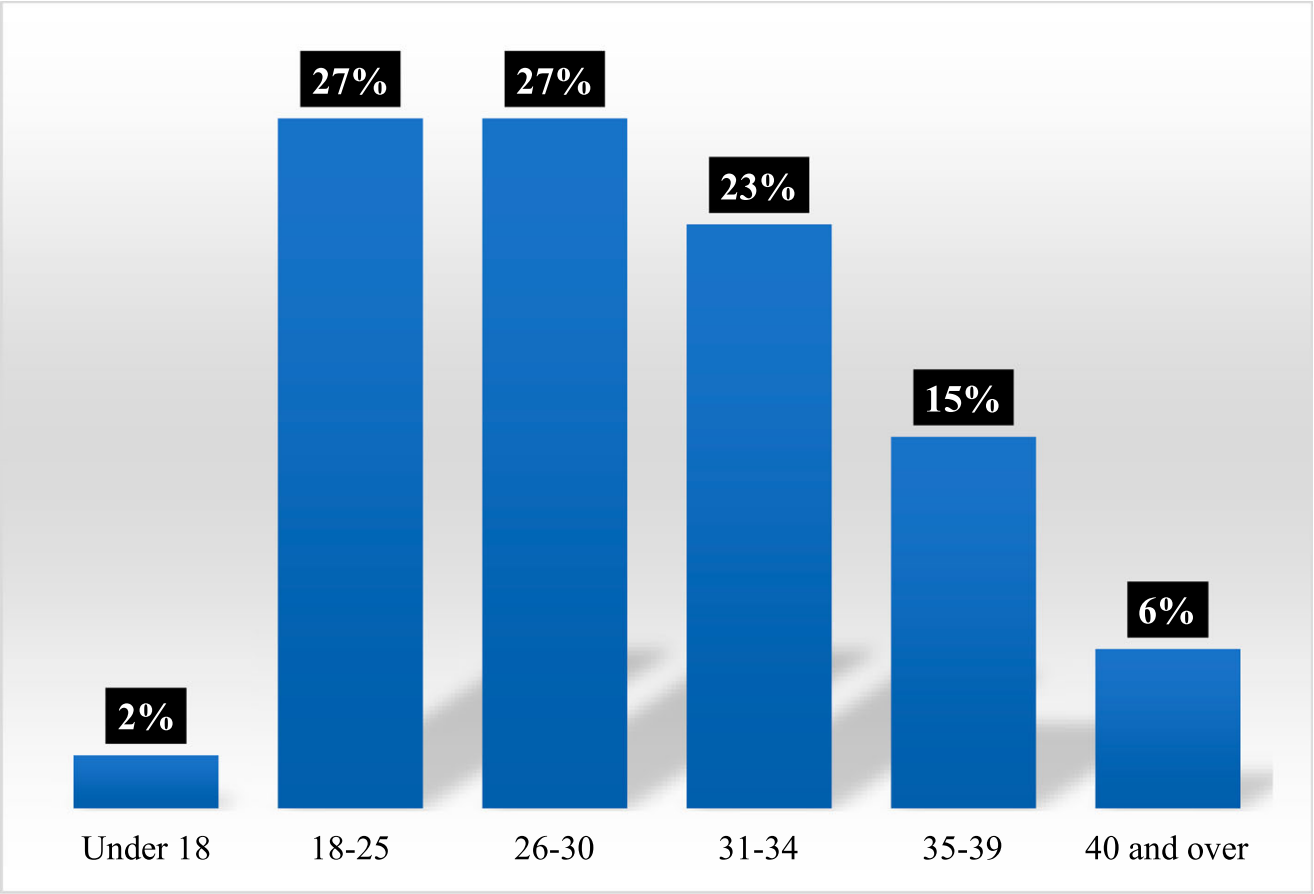
Age.

In these two seasons, the age of the birthing women also varied with some depicted as under 18 (2%), larger numbers represented as 18–25 (27%) and 26–30 (27%), and smaller numbers depicted as 31–34 (23%), 35–39 (15%) and over 40 (6%) (Figure 2). The age of new mothers in England and Wales in 2015 were recorded as 3.4% being under 20, 15.5% being 20–24, 28.4% being 25–29, 31.2% being 30–34, 17.3% being 35–39 and 4.2% being 40 and over (ONS, 2015). It is difficult to ascertain whether the representation of age maps accurately against national statistics for new mothers because our age groups differ from those of the Office for National Statistics (ONS). However, our study suggests that there is a slight over-representation of younger mothers in *OBEM*. O’Brien Hill (2014) argues that the show engages with contemporary debates on women’s age and reproductive and birthing choices. The distribution of age reflects the changing nature of birth in the U.K. as women are now having children later because they are expected to contribute to the labor market under the “new sexual contract” (McRobbie, 2009). Therefore, our data supports qualitative observations (Feasey, 2012; O’Brien Hill, 2014) that women’s social position and age is relatively diverse in *OBEM*.

Overall, whilst age and social class were slightly more diverse than expected, the diversity of women in *OBEM* was nonetheless incredibly narrow with the majority of women depicted as white, able-bodied, heterosexual, in a couple and working-class in these two seasons.

## Representing birth: ambiguity, absences and medicalization

### Ambiguity of birthplace and caregiver

Midwives are central to the U.K. version of *OBEM* (unlike the U.S. version) (Feasey, 2012; Horeck, 2016) reflecting the national maternity care context. The seasons analyzed here were filmed in Liverpool Women’s Hospital. Options for birth on the hospital site include a midwifery unit (MU) or obstetric unit (OU) (Liverpool Women’s Hospital, n.d.). English maternity policy states that women with a straightforward pregnancy should be offered the choice of giving birth at home, or in a MU or OU (NICE, 2014). In the absence of complications, women giving birth at home or in a MU will receive care directed by a midwife without medical input. Midwives provide the majority of care for women birthing in an OU, excluding cesarean section.

The content analysis aimed to code the representation of birthplace. No births took place in the home. However, we found it difficult to ascertain whether a birth took place in a MU or OU. Sometimes birth narratives shift between these sites, yet recognizing this relies on knowledge of birthplace options and the differences between them, which we might assume most viewers will not have.^4^*OBEM* occasionally uses signifiers of MUs (e.g. hospital signs), however mainly we—and, more importantly, most likely an audience member with no midwifery knowledge—found that the difference between the representation of MUs and OUs was ambiguous. Despite midwives being a focus of *OBEM*, the seasons offered a representation of MUs as indistinguishable from OUs, thereby rendering the specificity and difference in settings unimportant. The simultaneous visibility/invisibility of MUs in *OBEM* does political work. The majority of U.K. births happen in an OU; 13% of births occur outside OUs (National Audit Office as cited in Coxon, Chisholm, Malouf, Rowe, & Hollowell, 2017). The Birthplace in England Study (Brocklehurst et al., 2011), however, evidences that for healthy women with low risk pregnancies, planned birth in a MU is as safe as birth in an OU and involves fewer interventions. However, MUs are not available everywhere and where available are sometimes underused. In rendering MUs indistinguishable from OUs, *OBEM* misses an opportunity to represent different settings for birth and emphasize the available choices of birthplace for women in the U.K.

The research team also found it difficult to code the lead carer (midwife, obstetrician). This was because the show’s editing omits this information in favor of taking as central the individualized, emotional journeys of birthing mothers. Thus, despite the heightened visibility of midwives’ care in *OBEM* (Hamad, 2016; O’Brien Hill, 2014), the unclear role delineations between midwifery and obstetrics diminishes midwives’ role and responsibility in birth, reiterating a patriarchal history of obstetric dominance over midwifery (Witz, 1992).

### Birth position: women on their backs

**Figure 3.**
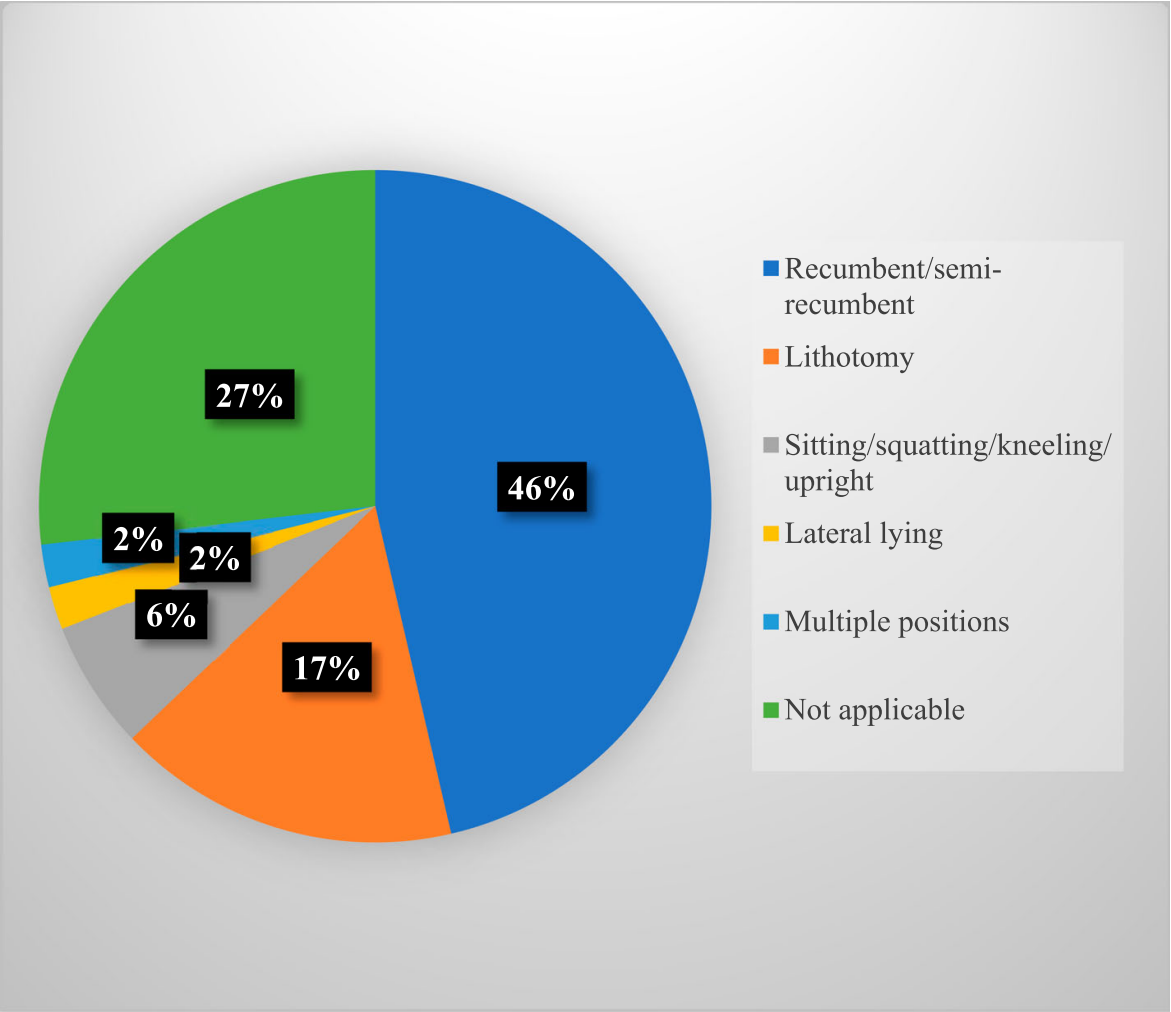
Birth position (second stage).

We coded the position adopted by women during the first and second stages of labor. During the first stage of labor, women were shown in various positions; women were shown in multiple positions (42%), recumbent/semi-recumbent (35%), lateral lying down (4%) or upright (2%). In the second stage of labor, women were mainly depicted in a recumbent/semi-recumbent position (46%) or in the lithotomy position (17%)^5^ (Figure 3). Only small percentages of women were shown in non-recumbent positions, such as sitting/squatting/kneeling (6%), lying down laterally (2%) or in multiple positions (2%).^6^ Overall, the majority of women (90%) pushing or having assistance before a baby is born were depicted on their backs in these two seasons. The visual centrality and repetition of the hospital bed or operating table that women lie down on in this stage also naturalizes the hospital ward/MU as *the* site for birth.

The representation of how women give birth raises interdisciplinary concerns. The repetition of this image represents women as passive (Morris & McInerney, 2010; Sears & Godderis, 2011) rather than active in birth. The image of women giving birth on their backs supports the discursive visualization of the western medicalization of birth within a patriarchal society, placing control with medical staff (Sears & Godderis, 2011). There are physiological consequences for women birthing on their backs. Birthing in a recumbent position is associated with detrimental falls in blood pressure and potential fetal compromise (Lawrence, Lewis, Hofmeyr, & Styles, 2013). By contrast, active and upright positions are associated with shorter labor, less intervention, less pain and greater satisfaction with birth (Priddis, Dahlen, & Schmied, 2012). Feminists have argued that birthing on one’s back is convenient for care providers but removes control from birthing women and have opposed requirements for women to give birth in such positions (e.g., the 1982 Birth Rites Rally, see Roberts, Tyler, Satchwell, & Armstrong, 2016).

### Interventions in birth: making the commonplace appear “normal”

Midwives and birth activists have expressed concern that televised birth over-represents interventions, medical emergency and surgical birth (J. Roberts et al., 2017). Our content analysis highlighted that, in these two seasons at least, most women were depicted giving birth vaginally, 58% without assistance and 15% with assistance, and 27% of women by cesarean section. This maps accurately against the different types of birth experienced by women in the U.K., whereby 60% gave birth without assistance, 13% with assistance and 27% by cesarean section (NHS Digital, 2016). RTV birth shows have been critiqued for reproducing ideologies around the medical model of birth that uphold technology and intervention into birth, and render women as inferior and passive (Morris & McInerney, 2010; Sears & Godderis, 2011; Tyler & Baraitser, 2013). Speaking of televised birth, Kitzinger and Kitzinger (2001, p. 61) argue that central to television’s birth mythology is the medical emergency, which “feeds the fears inherent in the dominant medical model of birth and […] conditions pregnant women to submit to its rituals.” Thus, one might expect an over-representation of cesarean birth, but our content analysis reveals a different picture, whereby representation of cesarean birth was in line with national statistics. Whereas some qualitative studies, and midwives and birth activists (see J. Roberts et al., 2017) have often focused on those interventions that are most highly dramatized, our analysis of these two seasons of *OBEM* suggests that other technocratic interventions are more prevalent in ways that serve to naturalize them as commonplace aspects of birth.

**Figure 4.**
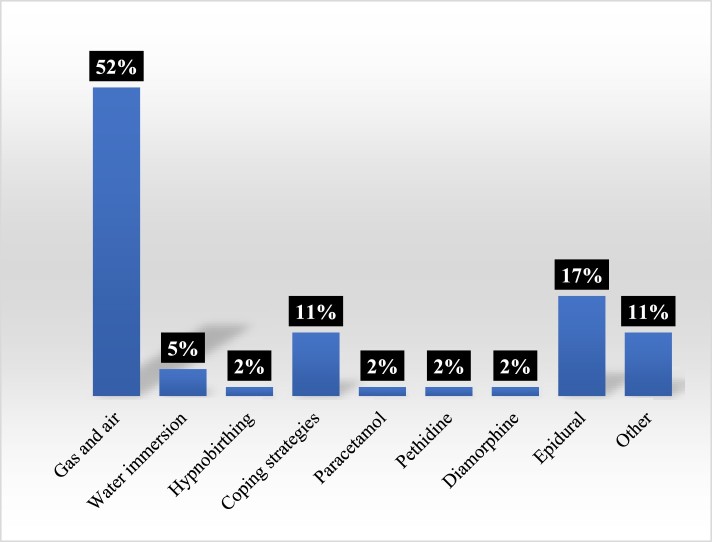
Pain relief.

**Figure 5.**
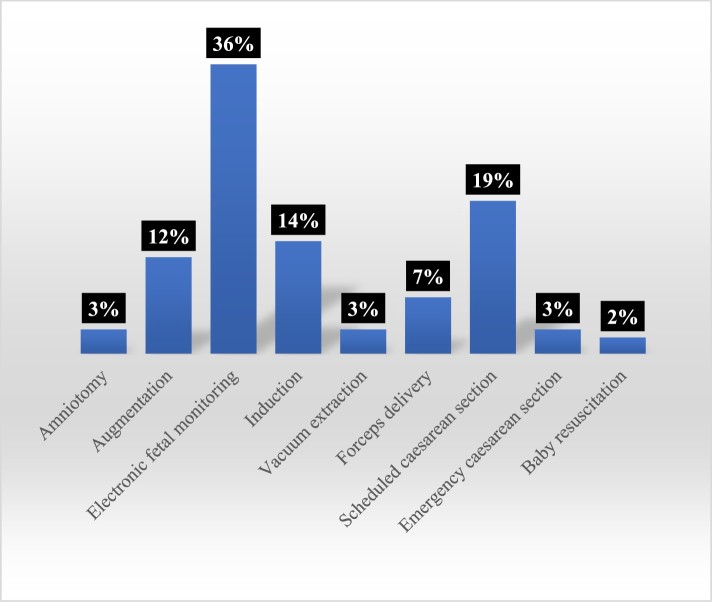
Procedures.

The content analysis highlighted that most births portrayed in these two seasons of *OBEM* featured pain relief (98%) or a procedure (77%). Of the births depicting pain relief, the most depicted methods were inhalational analgesia (“gas and air”) (52%) and epidurals (17%) (Figure 4). Of the births depicting procedures, the most common were electronic fetal monitoring (36%) and scheduled cesarean section (19%) (Figure 5). Unassisted vaginal births were therefore shown to have a high number of interventions, predominantly because of the representation of gas and air or electronic fetal monitoring. However, these interventions are not inevitable or uncontroversial. For example, some argue that intrapartum electronic fetal monitoring has minimal benefits for women with straightforward pregnancies and increases risks, and is associated with a cascading of birth interventions (Alfirevic, Devane, & Gyte, 2013). Furthermore, the use of monitoring sometimes restricts women to their beds, with the physiological consequences detailed above. In the context of televisual representation, it also contributes to gendered representations of passive birthing bodies.

Notes taken during the coding stage highlighted that in these seasons there was frequently no depiction of discussion about why gas and air or electronic fetal monitoring were introduced into the birthing process. Rather, these would appear on screen at some stage with an absence of information, context or explanation. These findings echo the U.S. context, supporting Morris and McInerney’s (2010) point that interventions are implicit in birth shows, but also raising questions about choice over interventions in labour care.

## The absence of information and choice over interventions

In the seasons of *OBEM* analyzed, depictions of information giving and facilitating choice around interventions were largely absent. We explored the representation of how decisions over procedures and pain relief were made; whether information was given about interventions, who requested interventions (women, partner or family) and the outcome of requests.

Overwhelmingly, information-giving was lacking in the episodes investigated. In 72% of births that featured a procedure, information about procedures was not depicted and in 73% of births that featured pain relief, information-giving about pain relief was absent. In 91% of birth stories depicting a procedure, discussions facilitating choice were not shown, a technology appeared on the screen without comment, or a procedure was undertaken without any discussion of options. In the other 9% of labors depicting a procedure, women requested procedures. The outcome of choice over procedures was not represented 91% of the time because choice discussions were largely unrepresented. Likewise, in 68% of births that featured pain relief, choice over pain relief was not shown and 21% of the time women were represented requesting pain relief. This meant that coding for the outcome of discussions of choice over pain relief resulted in 75% of the time there being no outcome to code for pain relief. Overall, there were very limited depictions of discussions of options or choices being made by women.

We interpret the depiction of information and choice with caution; our analysis can only determine what was aired in these two seasons and cannot speak to the conversations that may have happened during labor or the editing process. However, our analysis accords with Morris and McInerney’s (2010) quantitative analysis about the absence of information in U.S. RTV birth shows. This is significant as they (2010, p. 136) note, a “key way women can control birth is by choosing to consent to medical procedures only after receiving full information on their benefits and risks”. It also coheres with Jackson et al.’s (2017) conversational analysis of *OBEM* that found that healthcare professionals overwhelmingly introduce interventions to women with assertive phrases such as “we need to … ” or “we are going to … .”

Our analysis challenges a qualitative U.K. study finding that *OBEM* may be more informative than other birth shows (Feasey, 2012). *OBEM* could be understood as generally informative as a series, but it does not appear to provide detailed information of birth choices. In omitting representations of information giving, discussion of risks and benefits, and the exercise of informed choice, these interactions arguably remain outside of the mainstream discourse of birth in the U.K. context.

Choice is central to U.K. policy (among many other national contexts). *Better Births* (NHS England, 2016) recommends women should have genuine choices, underpinned by unbiased information and supported by healthcare professionals. Nonetheless, studies have noted the difficulties of achieving this in practice (e.g. Crossley, 2007; Malacrida & Boulton, 2014). Although choice is not only structured by technocratic, medicalized cultures of birth,^7^ childbirth movements have stressed that women’s choice and control in birth was fundamental to realizing women’s birthing autonomy (Crossley, 2007). The lack of birth information and choice depictions in these two seasons of *OBEM* potentially positions women as subordinate to the birth process, their bodies and those considered more able to make birth decisions.

## Conclusion

Our findings both corroborate and challenge existing research, opening up new lines of inquiry for research to understand the impact of the contemporary terrain of televised birth. There are limitations to our study, such as the small sample size of two seasons, the focus on one program and the geo-cultural specificity of the analysis presented hitherto. Our findings can only speak to the two seasons analyzed. Yet, by situating this content analysis in relation to a broader body of work on depictions of birth in RTV, we can begin to draw out some wider conclusions and future avenues of research.

Our analysis corroborates existing research from the U.S. context which suggests that there is an over-representation of white, heterosexual, able-bodied couples in contemporary mediated birth narratives, albeit with some variations in class and age. In these seasons of *OBEM*, a narrow representation of women as the subjects of birth intersects with wider representational inequalities around race, sexuality, disability and class. The visibility of certain groups, however, does not mean that they are always progressively represented in the birthing process. Our analysis supports existing textual analyses that highlight the dominance of the medical model of birth that overwhelmingly represents women as passive subjects, visualized through representations of women on their backs, with limited if any input in decision-making during labor. Our findings challenge the claim that cesarean birth and medical emergency are over-represented on RTV as the proportion of representations mirror national statistics. However, we noted the repetitive representation of less-invasive interventions such as gas and air and electronic fetal monitoring in ways that positions these as unremarkable and perhaps inevitable parts of the birthing process. Furthermore, our analysis underscores that the depiction of choice and information over interventions was lacking, although it is acknowledged that discussions and decisions about interventions do take place “off camera.”

We reflect that working in an interdisciplinary team, comprising cultural/media studies, sociology of health and midwifery, has been productive to tune our analysis to contemporary political issues. Illustrative of this is novel insights produced around the visibility of midwifery care and MUs in seasons eight and nine of *OBEM*. By coding specifically for birthplace and lead carer, this content analysis places the invisibility of MUs and the specificity of midwives’ roles in representations of childbirth on the agenda for future studies. At a time when U.K. midwifery and midwifery-led care are attacked under austerity (Asthana, 2017) and various birthplace options are under threat, we would argue that these representations are problematic. Thus, we echo Morris and McInerney’s (2010) suggestion that more dialogue between researchers, midwives, producers and women is needed to inform the televisual representation of birth.

*OBEM* attracts large audiences in the U.K. While popular, the way that birth is represented on this primetime program has raised important questions about birth politics amongst academics and the birth community. A quantitative approach has proved valuable to both support and challenge existing studies and further interdisciplinary research could usefully investigate the representation of birth across multiple seasons, genres and national contexts, as well as explore how such representations are made meaningful by localized audiences.

## Notes

1 However, in common with textual analysis, content analysis cannot facilitate broader extrapolations about how a program will be understood by audiences (Messenger Davies & Mosdell, 2006, p. 98) (see Skeggs & Wood, 2012).

2 Season eight aired July–September 2015 and season nine aired March–May 2016.

3 This subcategory differentiated between a couple and a single mother with the father present at the birth. Although women may have different birth companions, *OBEM* places birth in the realm of the heterosexual couple. We were interested in the different ways the heterosexual relationship may have been represented.

4 Coxon et al. (2017) note that OUs are considered the “normal” birth site in the U.K. with reasons ranging in existing literature from women considering this the safest option, to alternatives not offered or adequately explained by healthcare professionals.

5 Lithotomy is when a woman’s legs are in stirrups (Gupta, Sood, Hofmeyr, & Vogel, 2017).

6 As mentioned, if a woman had a planned cesarean, on the whole the first stage of labor position was coded as “not applicable,” resulting in 17% of cases being “not applicable.” If a woman had a planned or emergency cesarean, the second stage of labor position was coded “not applicable,” resulting in 27% of cases being “not applicable.”

7 O’Brien Hill (2014) argues that giving birth “naturally” is now a rite of passage to “good” motherhood as women must selflessly “suffer” for their child. Further, she argues that these paradoxes are represented in *OBEM*, whereby neoliberal and post-feminist ideologies are maintained as feminist ideas of choice and empowerment are blurred with anti-feminist ideas of disempowerment through the imposition of certain choices (see De Benedictis, 2017 for an extended discussion in relation to viewers and *OBEM*).
